# Stigma, lost opportunities, and growth: Understanding experiences of caregivers of persons with mental illness in Tamil Nadu, India

**DOI:** 10.1177/13634615211059692

**Published:** 2022-02-16

**Authors:** Mirjam A. Dijkxhoorn, Archana Padmakar, Joske F. G. Bunders, Barbara J. Regeer

**Affiliations:** 1 The Banyan Academy of Leadership in Mental Health, Vrije Universiteit Amsterdam; 2 1190 Vrije Universiteit Amsterdam

**Keywords:** caregiver experience, India, mental illness, personal growth, stigma

## Abstract

This study aimed to address gaps in understanding of the lived experiences of caregivers of persons with mental illness in low-income countries. It was conducted among caregivers of persons with mental illness making use of a free non-governmental clinic in and around Chennai, India. The study adopted a qualitative methodology, with semi-structured interviews and life history exercises (n = 29) and six focus group discussions with caregivers (n = 21) and mental health professionals and community-based workers (n = 39). The experiences of caregivers were analyzed in the framework of “The Banyan model of caregiving,” which identifies six phases. Major themes in caregivers’ experience were: embarrassment and losing honor; fear; awareness; stigma and social exclusion; and reduced social interaction and loneliness. Posttraumatic growth considered as the result of caregiver experiences was found to consist mainly of personal growth and focusing on positive life experiences. Lost opportunities particular to the context of Tamil Nadu were described as the inability to get married, obtaining less education than desired, and loss of employment. Siblings faced lower levels of burden, while elderly mothers experienced especially high levels of burden and lack of happiness in life. Caregiver gains were identified as greater compassion for other people with disabilities, resulting in a desire to help others, as well as increased personal strength and confidence. Understanding the nuances of the caregiving experiences over time can provide a framework to devise more fine-tuned support structures that aim to prevent reductions in social interaction and lost opportunities, and improve a sense of meaning, in order to assist caregivers to continue providing care for their relatives with mental illness in a context with scarce mental health resources.

## Introduction

In India, caregivers of family members with mental illness provide invaluable and irreplaceable support for an estimated 90% of persons with mental illness (Thara, 2004; Thara et al., 1994). This dependence on the family for care and support is a result of a lack of mental health services in India as well as close-knit and dependent family structures (Aggarwal et al., 2011; Faqurudheen et al., 2014). The presence of family support has been found to be protective against issues such as homelessness and dire poverty for people with mental illness (Gopikumar, Narasimhan, et al., 2015; Radhakrishnan et al., 2021), especially in contexts where mental health services are inaccessible or non-existent.

India adopted a well-thought-out and comprehensive mental health policy in 2014, which includes support structures for caregivers (Government of India, 2014). In practice, the policies are not fully adopted or are poorly implemented. India is a country with enormous cultural, socio-economic, and rural–urban disparities in medical and social services, and improvement is expected to be a lengthy process. Care for persons with mental illness falls upon family members, who lack necessary support structures since the needs of caregivers are given scant attention in clinical practice (Jagannathan et al., 2011). It is equally important to design culturally sensitive support structures for caregivers, taking into account the social relations and lived conditions that frame family life. Towards this, qualitative understanding of the lived experience of caregivers, both in terms of burden and personal growth, as well as their relationship, as it changes over time is required. This is precisely the gap that this study tries to fill, with a specific focus on urban and rural areas in and around Chennai, Tamil Nadu.

## Background

When faced with the reality of a family member with mental illness, caregivers experience both positive and negative changes in their life. The extent of the impact has been established in numerous studies, which have investigated the scale of burden in various domains (physical, social, financial, emotional) (e.g., Awad & Voruganti, 2008; Chan, 2011; Hsaio et al., 2017; Joy et al., 2017; Shah et al., 2010) or have compared burden for different illnesses (Hastrup et al., 2011; Nehra et al., 2005). Others have widened the scope to understand potential gains to caregivers (Aggarwal et al., 2011; Chen & Greenberg, 2004; Monyaluoe et al., 2014; Szmukler et al., 1996). The results of these studies point to demonstrable differences between high- and low-income countries. Studies in European and North-American settings identify issues such as lack of involvement of caregivers in the treatment process (Chen & Greenberg, 2004; Friedrich et al., 2014; van der Voort et al., 2007), the impact of deinstitutionalization (Awad & Voruganti, 2008; Blanthorn-Hazell et al., 2018), high financial burden (Ignatova et al., 2018; Perlick et al., 2007), difficulties related to dealing with the behavior of the relative (Blanthorn-Hazell et al., 2018; Perlick et al., 2007; Magliano et al., 2005) and emotional reactions of caregivers to the trajectory of mental illness of their relative (Dharmi et al., 2017; Dunkle et al., 2015; Karp & Tanarugsachock, 2000).

In the smaller body of work on caregiving in low- and middle-income countries (LMICs), the concerns focus on the involvement of other community members in care (den Hertog & Gilmoor, 2016; Monyaluoe et al., 2014; Seloilwe, 2006), families seeking out varying systems of treatment for the person with mental illness (religious, private, and government) (Azman et al., 2017; Faqurudheen et al., 2014; Jack-Ide et al., 2013; Monyaluoe et al., 2014; Quinn & Evans, 2010), religious coping strategies (Azman et al., 2017; Chadda & Deb, 2013), lack of locally available treatment (Chadda & Deb, 2013; Jagannathan et al., 2011), high levels of stigma and social exclusion (Chang & Horrocks, 2006; Jack-Ide et al., 2013; Thara et al., 2003), and financial circumstances limiting access to mental health services for the affected person (Addo et al., 2018; Chen et al., 2019; Quinn & Evans, 2010; Seloilwe, 2006).

### Effects of caregiving on families in India

Caregiver experiences are shaped by the cultural context, family structures, and societal perceptions of mental illness. This section focuses on caregiving in the Indian context, and explores the special contextual and cultural factors that impact caregivers’ lives.

#### Negative effects of caregiving: Caregiver burden and stigma

In many parts of India, stigma related to mental illness is a major concern, affecting persons with mental illness as well as their family members. *Courtesy stigma*, as described by Goffman (1963), also called *affiliate stigma* (Mak & Cheung, 2012), is an important aspect of caregiver burden. It refers to stigma experienced by someone who is associated with another person affected by stigma, such as someone with mental illness. Despite providing essential financial, physical, and emotional support to their relatives, many family members report experiencing disapproval and devaluation by society (Lauber & Rössler, 2007; Thara et al., 2003). The effects of stigma on caregivers, however, remain understudied in many LMICs, including India, and negative consequences for family members as a result of stigma have been less well understood (Koschorke et al., 2014; Lauber & Rössler, 2007; Loganathan & Murthy, 2008; Semrau et al., 2015; Tanaka et al., 2018).

In India, mental health stigma is related to lack of awareness about mental illness, as well as various religious and magical explanatory models for the causes of mental illness, such as demon possession and black magic (Lahariya et al., 2010; Padmavati, 2005). The incidence, predictors, and consequences of stigma include social ostracism, loss of relationships, and the inability to marry (Chan, 2011; Chien et al., 2004; Ergetie et al., 2018; Macleod et al., 2011; Mishra et al., 2012; Wong et al., 2018). The inability to marry can have severe consequences for caregivers, and particularly for women. The expectation of marriage for women is high in India, with almost 95% of women marrying by age 35 (Yeung et al., 2018). Arranged marriage, while on the decline, is still the most prevalent system of finding a spouse in India (Allendorf & Pandian, 2016). Caste endogamy is still highly prevalent (Allendorf & Pandian, 2016), and class, education, income, health status, and family structure of the future spouse are important considerations.

#### Positive effects of caregiving

Caregiving also has positive effects for the caregiver, including personal growth. Models of post-traumatic growth view the negative effects of traumatic experiences as necessary for growth to take place (Shakespeare-Finch & Copping, 2006). The relation between caregiver burden, positive aspects, and personal growth has been conceptualized in several models. For example, Szmukler et al. (1996) developed a model that includes the positive aspects of caregiving. This “experience of caregiving” model goes beyond the commonly used “stress-burden-coping” model, which focuses on negative aspects of caregiving, and includes two areas of gains: “positive personal experiences” and “good aspects of the relationship.”

#### Posttraumatic growth

Posttraumatic growth is defined as “positive psychological change experienced as a result of the struggle with highly challenging life circumstances or traumatic events” (Calhoun et al., 2000, p. 1). Several studies show that the concept of posttraumatic growth is relevant not only for people experiencing traumatic events themselves, but also for caregivers (Cormio et al., 2014; e.g., Cadell & Sullivan, 2006; Hallam & Morris, 2014; Thombre et al., 2010).

Theories of posttraumatic growth are based on core assumptions about the self, general benevolence, and the meaningfulness or predictability of events (Splevins et al., 2010). When unexpected or unanticipated events disrupt life, one's worldview and anticipated life path may need to be revised (Triplett et al., 2012).

Analyzing posttraumatic growth in specific cultural contexts is highly important, since posttraumatic growth is intrinsically linked to the sense of “self” of a person. In more collectivistic or interdependent societies such as India, the “self” is generally more defined in relational terms linked to family members and others in society, rather than the individual being considered the primary entity of consideration of growth (Splevins et al., 2010). Behavior in such societies is driven by the goal of upholding expected social norms as a means of maintaining social harmony (Splevins et al., 2010). In this context, not reaching society's expected milestones and adjusting one's life path can be considered a life major disruption and a cause of great distress to the individual.

A majority of the literature on posttraumatic growth, however, is based in western and high-income settings, where growth is considered an individual process. For instance, a framework for posttraumatic growth, contextualized by Shakespeare-Finch and Copping (2006) for the Australian context, comprises the concepts not only of “compassion” and “focus on life's positives,” but also of “personal strength” and “effortful reinvention of self.” In a more collectivistic society, like India, the self and personal growth may acquire a different meaning and urgency, which we will examine in this study.

### Aims of the study

Considering the knowledge gaps related to the social and cultural aspects of caregiver experience in India, we aimed to investigate the lived experience of caregivers of people with mental illness in a low-income setting, across the stages of their care journey, with particular focus on stigma, loneliness, lost opportunities, and caregiver growth.

## Methods

### Setting and population

The study was conducted among caregivers of people with mental illness in a rural (Thiruporur block in Kancheepuram district) and urban area (Chennai) of the South Indian state of Tamil Nadu (TN). In social and economic terms, TN is often considered to be an outlier among states in India. It has a population of 72.15 million people, with a sex ratio of 996 females per 1,000 males, which is well above the national average of 943 females per 1,000 males (Government of India Census, 2011). TN's literacy rate was 80.09% in 2011, which is higher than the national literacy rate of 74.04%, and the poverty headcount ratio at 11.71% is lower than India's rate of 21.9% (Government of India National Sample Survey, 2011–2012). While not at the same dramatic levels as certain states in North India, son preference and daughter aversion was observed in rural areas, due to drastically falling fertility rates and increased dowry demands over the previous decades (Diamond-Smith et al., 2008; Pande et al., 2012). In general, TN is a patriarchal society, which manifests in division of labor in the household and workforce (Government of India, 2014; Keiko, 2011), patrilocal practices, as well as domestic violence patterns (Chokkanathan, 2012).

According to the National Mental Health Survey of India 2015–2016 (NIMHANS, 2016), TN has reported a higher suicide rate than the national average, namely 23.4 per 100,000 population versus 10.6 for India. TN also reported a high prevalence of mood disorders and depressive disorders (4.62%) compared to the national prevalence of 2.8% (NIMHANS, 2016).

Mental health services are available at the Kancheepuram and Chennai government hospitals, at the Institute of Mental Health in Chennai, and at private clinics. Despite relative proximity to Chennai, parts of Thiruporur block are remote and not serviced by public transport, thereby making access to health care facilities expensive and time-consuming.

Several mental health non-governmental organizations (NGOs) are operational in Chennai and Kancheepuram district, offering services ranging from clinical treatment and community awareness to vocational training and advocacy. Limited long-term care facilities are available in Chennai and Kancheepuram district, but they charge high rates, which are unaffordable for many of the families included in this study.

The caregivers who participated in this study made use of the free outpatient clinics provided by The Banyan, an NGO. The aim of the organization is to offer comprehensive care by a multi-disciplinary team, including medical treatment, emergency and long-term care, psychological services, and employment and skills development, as well as facilitating access to social protection measures. In 2018, 959 (403 male and 556 female) people accessed the rural clinics and 1,323 (698 female and 625 male) accessed the urban clinics.

### Study design

This study used a qualitative methodology, with semi-structured interviews, life history timelines, and focus group discussions (FGDs) (Padgett, 2012), in order to provide specific information that could provide an in-depth understanding of the lived experiences of caregivers in TN. In order to examine how caregivers give meaning to the experience of caregiving, the phenomenological perspective was used. In this perspective, human experiences are viewed as dynamic, complex, and continually moving (Gray, 2018). The phenomenological perspective also provides opportunities to consider the cultural context as an influence on the human experience. This approach suited the aim of this study, which included understanding the cultural meaning of being a caregiver of a person with mental illness in a low-income family in India, and the changing nature, in both positive and negative terms, of caregiver experiences over time.

Semi-structured interviews were conducted with 29 caregivers using an interview guide prepared by the researchers. Each interview lasted approximately 1.5 to 2 h; interviews were conducted over a span of two months. Questions pertained to financial, social, and emotional aspects of caregiver burden; stigma and social life; positive aspects of caregiving; relationship with the relative with mental illness; the trajectory of illness; requirements from the relative for care; help from other relatives or community members; treatment sought; long-term care plans; and support structures required. The guide was piloted with five interviews, after which the researchers discussed the interview guide and restructured questions that were misunderstood and added items for additional information.

Life history timelines with positive and difficult experiences for the period of the mental illness of the relative were prepared with the 29 participants. Life history timelines have been shown to be an effective interview technique to help participants to remember past events (Adriansen, 2012). Caregivers were invited to identify positive experiences and to place them above the appropriate time frame, while negative experiences were placed below the line. The events in the timelines were then used as a starting point for additional questions. Appreciative Inquiry (Regeer et al., 2009; Whitney et al., 2019) was chosen as a result of ethical considerations related to the chosen methodology. The caregivers were asked during the interviews and FGDs to share and reflect on how they developed coping mechanisms and found strength within themselves and their communities. This aim was not only to gain a multi-faceted understanding of the caregiver experience, but also to provide the caregivers an opportunity to share positive experiences and strength derived from their experiences, as opposed to singularly reliving distressing memories; for example, caregivers were asked to reflect on how they had changed as a person, in either positive or negative ways.

Based on the interviews and life history timelines, the authors developed a model of phases of caregiving, which is described elsewhere (Dijkxhoorn et al., 2018) and in the data analysis section below. The Banyan Model of caregiver experiences consists of six phases, from the initial symptoms to long-term growth, including corresponding support needs in each phase. Following this, six FGDs were conducted with caregivers (n = 21), and with mental health professionals and grassroots workers (n = 39). The aim of the FGDs was to verify the accuracy of the phases of caregiving and to understand the feelings and experiences of caregivers in each phase, since this was not included in the interviews.

### Data collection

For the interviews and FGDs, purposive sampling (Gray, 2018) was employed to select participants from caregivers making use of The Banyan's outpatient mental health clinics. Maximum variation sampling (Padgett, 2012) was chosen to include four types of caregivers of people diagnosed with mental illness in the interviews, in order to gain a variety of perspectives: a) parents; b) adult children; c) spouses; and d) siblings. All participants had been caregivers for at least two years and lived with the relative. All relatives with a mental illness of the caregivers participating in the interviews were female. The aim of the first study phase was to understand the experiences of caregivers of women exclusively. After the development of the model of caregiver experiences, caregivers of men were included in the FGDs to elicit a broader perspective of caregiver experiences and to make the model applicable to caregivers of persons of both sexes. The interviews were conducted in the homes of participants, or at a private space at The Banyan clinic.

After the interviews and the development of the model of caregiver experiences, six FGDs were conducted with caregivers of men and women diagnosed with mental illness making use of The Banyan's Urban Mental Health Programme (parents and spouses (n = 12)) and Rural Mental Health Programme (siblings and adult children (n = 8)), and with mental health professionals and NALAM^1^ community workers from urban (n = 10 and n = 10) and rural areas (n = 8 and n = 12). During the FGDs, the investigators explained the phases of the caregiving model, following which participants created charts in small groups of two or three participants that detailed their feelings and experiences during each phase, which were then discussed in the larger group.

The first two authors conducted the interviews and FGDs. The first author (MD) is a social anthropologist from the Netherlands who has lived in India since 2006. She has a basic understanding of Tamil. The second author (AP) is a clinical psychologist from Tamil Nadu and speaks Tamil fluently. The last two authors (JB and BR) are from the Netherlands and have spent extended periods of time in LMICs as researchers.

### Data analysis

All interviews and FGDs were audio recorded and professionally transcribed, and if not in English, translated from Tamil to English. In addition, notes were taken during each interview and FGD. Inductive coding was employed by the first two authors and a codebook was developed using Dedoose software.^2^ After creating codes individually for five interviews, the authors compared codes and discussed the final codebook. Thematic analysis was conducted in order to identify common themes that caregivers indicated were most important in their experience (Gray, 2018). Relevant codes were then assigned to each theme after discussion between the authors, and the themes were examined in more detail to describe the most important aspects of each theme (see Table 1). Data saturation was discussed.

The charts prepared in the FGDs with feelings and experiences of caregivers in each phase were tabulated and items with the highest incidences were included in the description of the phases, in addition to identification of codes and quotes that corresponded with each phase. Frequently recurring themes that spanned various phases were identified from the data.

**Figure 1.**
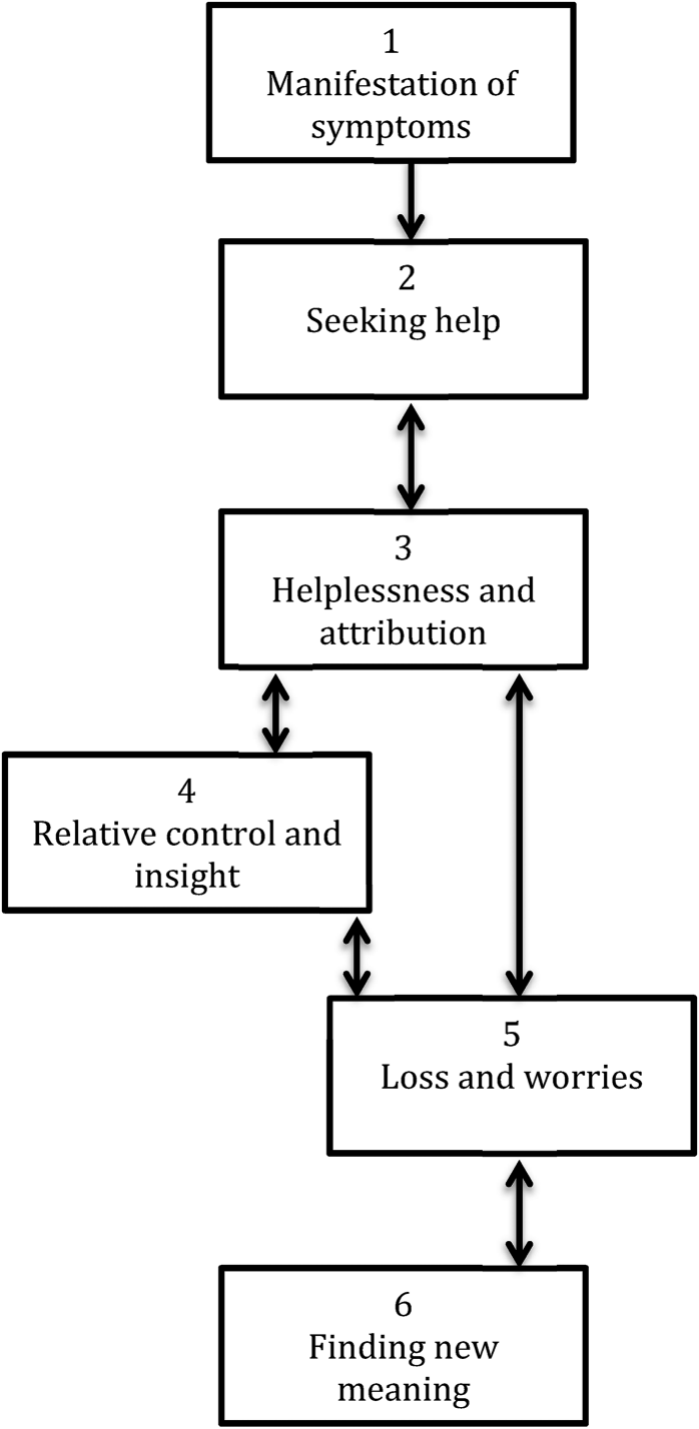
The Banyan model of caregiving experiences.

The results were analyzed using a framework developed by the authors named “The Banyan model of caregiver experiences” (Dijkxhoorn et al., 2018), which emphasizes the different phases that caregivers experience over time. Six phases were identified, as shown in Figure 1 and described in Table 3: 1) Manifestation of symptoms; 2) Seeking help; 3) Helplessness and attribution; 4) Relative control and insight; 5) Loss and worries; and 6) Finding new meaning. The phases do not necessarily follow a linear succession. Moreover, not all caregivers experience all phases, and certain phases may never be left behind.

#### The Banyan model of caregiver experiences (from Dijkxhoorn et al., 2018)

This model of caregiver experiences is a useful framework for analyzing changes in experiences over time, as well as identifying common themes across phases. Whereas the purpose of the first study (Dijkxhoorn et al., 2018) was to construct and present a phased model of caregiving experiences, the purpose of this study is to gain in-depth insight into the lived experiences of caregivers, how experiences change over time, which themes are common across phases, and how experiences relate to each other. We will also use the posttraumatic growth framework developed by Shakespeare-Finch and Copping (2006) to examine whether and how the four domains identified in their model are described by caregivers in this study and/or whether other areas of personal growth are relevant in the Indian context.

#### Ethical considerations

The Institutional Review Board of The Banyan approved the study (Approval number: EEC-2015-2). The purpose of the study was explained in writing or orally to participants in Tamil or English, and written consent was obtained after the option of non-participation or refusal to answer questions was explained to participants. If a participant was non-literate, their thumbprint was obtained and a witness signed. All data were anonymized during data analysis and the hardcopies of interview notes were stored in locked cupboards.

## Results

The characteristics of caregivers, mental health professionals, and community workers who participated in the interviews and FGDs are presented in Tables 2 and 3.

**Table 1.** Example of coding and thematic development process.

Original text	Code	Theme
I think about my marriage and the need for a companion in my life who would take care of me. But people say that it is not possible in my life. In that case I feel constantly upset and depressed.	Life changes because of caregiving Psychological problems of the caregiver	Lost opportunities

**Table 2.** Characteristics of caregivers.

	Interviews (n = 29)		Focus group discussions (n = 21)	
Age (years), Mean (SD)			52.4 (13.5)	
Caregiver	48.8 (17.6)			
Relative	46.1 (11.8)			
	n	%	n	%
Sex of caregiver				
Female	18	62.1	13	61.9
Male	11	37.9	8	38.1
Sex of relative				
Female	29	100	18	85.7
Male		0	3	14.3
Type of caregiver				
Parent	8	27.6	5	23.8
Spouse	6	20.7	8	38.1
Sibling or sister-/brother-in-law	8	27.6	6	28.6
Adult child or son-/daughter-in-law	7	24.1	2	9.5
Education of caregiver				
No education	3	10.3	1	4.8
Up to 5 years	7	24.2	2	9.5
Up to 8 years	6	20.7	7	33.3
Up to 10 years	7	24.2	9	42.9
Up to 12 years	0	0	1	4.8
Higher education	6	20.7	0	0
Unknown			1	4.8
Marital status (caregiver)				
Unmarried	5	17.2	1	4.8
Married	14	48.3	17	81.0
Widowed	8	27.6	3	
Divorced or separated	2	6.9		14.30
Duration of caregiving (years)				
Mean (SD)		14.2 (9.49)		14.0 (9.96)
Range		2–40		2–39 years

**Table 3.** Characteristics of mental health professionals and community workers.

Participant focus groups	Mental health professionals and community workers (n = 39)	SD
Location		
Rural mental health programme	19	
Urban mental health programme	20	
Occupation		
Social worker	4	
Psychologist	2	
Senior community worker	2	
*NALAM* worker	21	
Nurse	2	
Occupational therapist	1	
Vocational trainer	2	
Health care worker	2	
Mean years of experience	3.6	(SD = 3.09)
Mean number of clients assisted	241	(SD = 259.93)

All participants in the study were from low- or middle-income households (with monthly family incomes of INR 1,500 (approximately US$21) to INR 15,000 (approximately US$210).^3^ The family income was self-reported and not independently verified.

When analyzing the experiences of caregivers, four themes were identified, namely embarrassment and losing honor; fear; awareness, stigma, and social exclusion; and reduced social interaction and loneliness. The themes and manifestations in the different phases are shown in Table 4 and explained below. We describe how embarrassment, fear, and stigma manifest in the daily life of caregivers, and how these issues lead to reduced social interaction.

**Table 4.** Changes in themes over time in the Banyan model of caregiver experiences.

	**Phase 1:** **Manifestation of symptoms**	**Phase 2:** **Seeking help**	**Phase 3:** **Helplessness and attribution**	**Phase 4:** **Relative control and insight**	**Phase 5:** **Loss and worries**	**Phase 6:** **Finding new meaning**
	Changes in behavior and early symptoms of mental illness are observed in the relative. The caregiver faces uncertainty about the cause and about places for treatment of the symptoms.	The caregiver aims to seek treatment and help for the relative's symptoms, often at various types of treatment facilities (faith-based, government, private).	When seeking help does not yield the expected level of recovery, caregivers experience helplessness. Attribution of the causes of the mental illness to experiences in the past often occurs as a coping strategy.	When symptoms stabilize with treatment and caregivers learn how to recognize and deal with the changed behavior of the relative, they experience a sense of mastery and relative control, as much as is feasible with the unpredictability of the course of mental illness.	Caregivers recognize opportunities lost in their own life, due to assuming caregiver duties, as well as in the life of the relative due to mental ill health. Caregivers also worry for the safety of the relative, as well as who will provide care after they are no longer able to.	Caregivers can find new meaning in life as a result of providing care, in the form of helping others, being able to help their relative achieve independence or employment, or finding employment as a mental health worker.
**Embarrassment & losing honor**	Embarrassing behavior of the relative		Relative or caregiver not adhering to society's expectations (e.g., marriage, education, jobs)		Relative or caregiver not adhering to society's expectations (e.g., marriage, education, jobs)	
	Perceived loss of honor when the relative wanders away and the caregiver feels judged by the neighborhood					
**Fear**	Fear that the relative is possessed by demons or evil spirits					
	Fear of the relative when he or she is violent					
	Fear of the future and whether a cure is possible			Fear of the future and who will take care when the caregiver is no longer able to		
	Fear for the safety of the relative					
**Awareness, stigma, & social exclusion**	Expectations of stigma in the community	Lack of awareness of treatment facilities	Stigma attached to seeking counseling by the caregiver		Supportive relatives, friends, & communities help to alleviate feelings of loss	Caregivers develop more sensitivity towards people with disabilities
		Stigma attached to seeking treatment				Caregivers want to help other caregivers through peer groups
						Caregivers desire to spread awareness and reduce stigma
**Reduced social interaction and loneliness**	Nobody to share the caregiver's worries about changes in the relative with	Nobody to advise about treatment facilities	Nobody to share feelings of helplessness with	Caregivers start sharing experiences of the mental health issues of the relative with others	Mourning the loss of contact with friends and relatives	Create new social ties with other caregivers or people in the community

### Embarrassment and losing honor

Caregivers expressed apprehension about losing honor (மரியாைத – *mariyathai* in Tamil) in phases one (manifestation of symptoms), two (seeking help), and five (loss and worries). In particular, certain public behavior caused embarrassment, such as verbal abuse of the caregiver or others, shouting, or going outside naked: “[S]he was asking everyone for money, which is very embarrassing. Sometimes when she gets angry, she verbally abuses and even beats us” (Sister, 50 years old); “She used to dance, with her clothes in disarray. The house owner asked us to vacate the house, because if anything happens we might blame the house owner” (Husband, 57 years). Loss of rented housing as a result of having a family member with mental illness was a common occurrence for many families, following complaints by neighbors or worries by house owners about their property.

In addition, when the relative did not meet normal social expectations, such as marriage, having children, employment, and education, or if the caregiver's expectations for the relative were not met, the family perceived a loss of honor. Being married is an important social status, as it is considered necessary to fulfil one's social and religious duties (*dharma*). Therefore, the inability to marry as a result of mental illness can itself be a form of stigma. The family as a whole can also experience loss of honor when siblings of the relative with a mental health issue become less eligible for arranged marriages, because of stigma, worries about the hereditary nature of mental illness, and having to accept caregiving duties. Some caregivers shared that having a person with mental illness in the family was considered a sign that the family as a whole had bad luck (*thurathishtam* – துரதிர்ஷ்டம் in Tamil) or was surrounded by bad spirits (*ketta katru* – ெகட்ட காற்று).

Wandering away from home and getting lost or walking around the roads the whole day were also mentioned as causes for loss of honor, because others in the community consider this a sign of inadequate care. Honor was not just considered an individual issue by caregivers; actions of one family member affect others in the family too. Family members shared that the need to discipline the actions of the person with mental illness was not only in the interest of the individual but was aimed at maintaining the honor of the whole family. Loss of honor had potentially devastating consequences, such as social isolation and loss of relationships, as is described below, in addition to potential loss of housing.

### Fear

Caregivers experienced fear and worries in almost all phases, namely one (manifestation of symptoms), two (seeking help), three (helplessness and attribution), four (relative control and insight), and five (loss and worries). Caregivers expressed fear of the relative being possessed by demons or evil spirits in the first and second phases, since they did not know what caused the symptoms. Signs of mental illness are often attributed to demon possession and black magic in TN.

In the first phases (1–3), some caregivers shared being fearful of the relative when s/he is violent. Caregivers also experienced fear of the future and whether the relative would ever recover, fearing the worst. The safety of the relative is a constant worry. Caregivers of female relatives worried about sexual assault when they were alone at home or out on their own: “Safety is a major concern. We are scared to leave her alone anywhere, because she is a woman” (Sister-in-law, 29 years old).

In the later stages (4 & 5), caregivers experienced fear of the future and uncertainty about who would take care of the relative when they were no longer able to. Caregivers often had no plan for who would take over in their absence, especially if there were no close relatives or if relatives had disengaged. Some caregivers expressed the hope that an NGO like The Banyan would take care of the relative and some left the future up to fate or God's will, without having concrete plans for an alternative arrangement: “After me, I don’t know who will take care of my wife. My son and future daughter-in-law may not take care of her. Because we are not able to say how people will change in the future” (Husband, 70 years old).

### Awareness of mental illness in the community, stigma, and social exclusion

Due to stigma and lack of awareness around mental health, caregivers often did not share their struggles with other family members for fear of ostracization. Nevertheless, they longed for community acceptance, as one daughter shared: “If people understood that she is different from others, but they do not need to be scared or ignore her, life would be much easier for me” (Daughter, 32 years old). Lack of community acceptance was evident in how a mother's illness affected her son, as recounted by his father: “My son is the most affected person. The people around keep talking to our son about our state and tell him that we are sinners and that is why we are being punished like this” (Husband, 56 years old).

### Reduced social interaction and loneliness

Reduced social interaction, fear of social situations, and loneliness were dominant themes for caregivers, as also described by community workers and professionals. Loneliness could be a result of a breakdown in family relations or, more subtly, of ceasing to receive invitations to family functions or no longer receiving visitors at home. At times, caregivers exhibited signs of self-stigma by avoiding social gatherings, as explained by one son: “My relatives do invite me to celebrations. But if we go there, they might talk about mother, so I do not go to such programmes” (Son, 31 years old).

In other cases, they missed social events because of caregiving duties, which could lead to loss of relationships:

I explain to my friends about my wife's condition and tell them that she is psychologically affected and that is the reason I will not be able to come to the function; they understand. Even if I go to any function, I return early. (Husband, 57 years old)

Almost all caregivers interviewed spent the entire day with the relative, except when going to work or on an occasional outing. A mother explained: “I spend the entire day with her attending to all her needs. I cook for her, boil water for her bath. She goes on asking for food, which I make for her” (Mother, 55 years old). In many cases, the relative preferred the caregiver to be at home at all times, and in some cases there were concerns of seizures or potential problems with neighbors that prevented the caregiver from leaving the relative alone. This became a time-consuming activity, as lamented by one daughter: “I spend the whole day, 24 h, with her. I put a chair next to her and sit down” (Daughter, 38 years old).

The claim on the caregiver's time was related to the need for them to keep the relative happy and to prevent violent outbursts or mental health relapses. While this might not be in the best interests of the relative, because independence is not fostered, caregivers felt that it was necessary to keep the peace. In addition, caregivers, and in particular female caregivers, shared that it was their duty to do whatever necessary for their relative, even if that meant putting their own lives on hold.

Loneliness was also voiced in the context of receiving family support. In the first three phases, caregivers described how they felt lonely in the process of caregiving. “Nobody to help,” “nobody to guide,” and “nobody understands the mental strain” were frequently used phrases. The feeling that they are solely responsible for finding successful treatment and the financial resources for it, without having information about mental illness or sources of support, caused them distress. Due to lack of awareness about the nature and sources of treatment for mental illness among the general public, extended family members of caregivers were unable to provide support in this area, as were most community health personnel and village leaders.

However, not all caregivers voiced loneliness. Some did not lose relationships with family members or friends and said that their social life was not affected. In some cases, the relative with a mental illness was the only person in the family to face stigma: “Everything is normal. We go to all functions and family members come home to visit. Sometimes she also comes with us” (Brother, 30 years old).

### Lost opportunities

Lost opportunities took various forms for different kinds of caregiver. We will first describe the experiences of adult children, since their life trajectory seemed invariably affected. Reasons included interrupted education, having to take care of themselves from a young age, or that they had not been able to get married. In a country where arranged marriages are common, finding a spouse, in particular for female caregivers, was problematic. The first reason was the older age of the caregivers when they initiated the search for a spouse, since they had spent their earlier years providing care. Mid- or late 20s is considered an advanced age for marriage for women by many. After this, finding a spouse is not impossible, but it poses more challenges. Secondly, having a relative with mental illness carries a stigma that affects all family members. Lastly, in the patrilocal kin structures in TN and most other states in India, women are expected to live with the husband's family after marriage and prioritize the family’s needs over their own. In this scenario, finding a spouse who takes full responsibility for a mentally ill mother-in-law can be nearly impossible.

Adult daughters shared their feelings about their inability to marry. For example:

I think about my marriage and the need for a companion in my life who would take care of me. But people say that it is not possible in my life. In that case I feel constantly upset and depressed. (Daughter, 26 years old)

Another daughter shared that she had lost out on marriage opportunities:

I have got many proposals [for an arranged marriage], but I have told them that I will get married only if I can keep my mother with me. Another problem is that I am getting older and there is no need for me to get married. Now my thought is only about my mother and brother and I will lead my life by taking care of them. (Daughter, 38 years old)

Disrupted education was another lost opportunity. Some adult children were forced to abandon the education of their own children because of financial difficulties: “Because we have a lot of debt for my mother's medical expenses, my children could not study after 12th standard, and they had to start working” (Daughter, 37 years old); “Because of this, our sons had to change their school from private to government schools” (Sister, 30 years old).

Moving frequently, because of the mother's illness, also affected the children's education: “My mother used to come to the school and create problems, because of which we had to move to a village from the city, where the education was not as good” (Daughter, 25 years old).

Many caregivers lost their jobs as a result of being a caregiver, either because their presence was required at home or as a result of taking time off to seek treatment. In other cases, the relative created embarrassing situations at the caregiver's workplace. A mother explained: “I lost my job, because my daughter behaved badly when I was there” (Mother, 59 years old). Two husbands pondered on their change in life plans as a result of losing employment. For example: “Sometimes I feel bad, because I used to have a decent income and a good life. Now I have lost everything, because I have to take care of my wife” (Husband, 67 years old). Another husband discussed his lost opportunities:

I had plans in my life about what kind of work I wanted to do and how to live. I always wanted to buy a house. But when my wife fell sick, I had to constantly look after her and search for her when she wandered away. So I don’t think I will be able to buy a house. (Husband, 57 years old)

### Caregiver growth

Even though caregivers most frequently described aspects of burden, and some caregivers reported no gains at all, positive aspects and perceived personal growth were also shared when asked about during the interviews, life history timelines, and FGDs. We will describe these positive aspects in the framework developed by Shakespeare-Finch and Copping (2006), which consists of the domains of personal strength, focus on life's positives, compassion, and effortful reinvention of self.

Increased personal strength and confidence, as a result of providing care and facing difficulties in life, was an aspect of growth often mentioned by caregivers: “[Being a caregiver] has made me strong, because I had to overcome a lot of problems. I feel good when I think about what I was able to do for my family” (Daughter, 37 years old); “I have more patience now, and I am more thoughtful about the way I do things in life” (Sister, 36 years old).

Caregivers focused on positive aspects of caregiving by appreciating praise or being able to learn from mistakes made by their relative. Two daughters shared their experiences:

Many relatives praised me at a recent family celebration for the way I take care of my mother. They said she looked much better than before and that it is admirable that I am able to handle her. That made me very happy. (Daughter, 38 years old)

[My mother's] life has been a big example for me, because I now know how not to lead my life. (….) She got severe depression, because my father betrayed her and spoiled her life. So I do not want to choose a wrong person like my father and I want the support of my family when I choose someone to marry. (Daughter, 26 years old)

These aspects of perceived personal growth and strength linked to adverse experiences indicate the building of resilience among some caregivers. Several of the caregivers referred to resilience as a necessity to survive being the sole caregiver for their relative. Personal growth and resilience were positive side effects of an otherwise distressing situation.

Another positive aspect of growth was strengthening of the relationship between caregiver and relative. We observed that caregivers in this particular sample (who had continued caregiving responsibilities) also showed love, affection, and a sense of responsibility as a constant drive for continuing. The main reason why most caregivers took care of and did not abandon the relative was because they felt love and affection for them. In addition, a strong sense of familial responsibility was observed, and caregivers considered providing care as natural and expected, instead of an extraordinary altruistic effort, despite the often great cost to their personal lives. Siblings and adult children emphasized that their parent had stressed the importance of being a caregiver over the years. Most spouses considered caregiving a normal consequence of being married and would have expected their spouse to do the same if the situation were reversed.

Caregivers also found that they had more compassion for and desire to help others in similar situations: “When I see someone in a similar situation, I try to speak to them and tell them to get treatment as soon as possible” (Brother, 51 years old). A husband shared: “While we are waiting at the clinic, I talk to new families and try to answer their questions. I like to help others, because we all had so many questions when we first came to the clinic” (Husband, 58 years old).

Reinvention of self was the domain that was least discussed by caregivers. During the interviews we found that many caregivers expressed difficulties with reflecting on how caregiving affected their sense of self and how they had changed as an individual. Most caregivers in the study live in poverty and are struggling to survive and manage their daily lives. In those circumstances, expending efforts on personal growth was not a priority. In addition, many described changes in terms of the impact on the whole family, not on them as individuals.

Nevertheless, some caregivers were able to reinvent themselves and their careers by obtaining jobs in mental health, as nursing assistants or community outreach workers (see also Dijkxhoorn et al., 2018): “I have learnt a lot about mental stability and psychiatric problems by observing my mother. Now I work as a nursing assistant with people with mental illness. The personal experiences with my mother helped me in this job” (Daughter, 26 years old).

**Figure 2.**
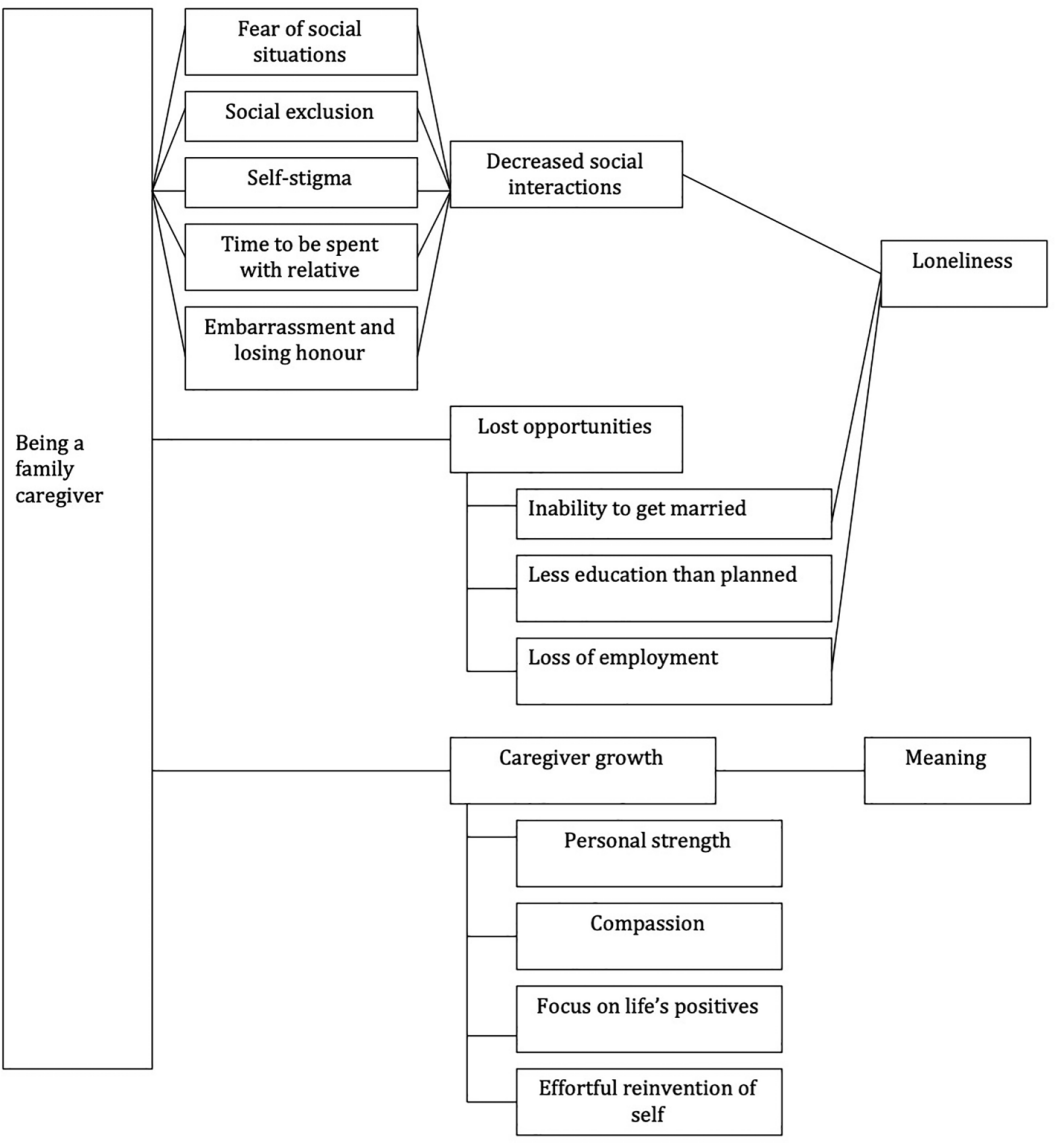
Links between caregiver experiences.

Although caregiver experiences differ among individuals and families, we found common themes across phases. We also found that these themes are highly intertwined. Many of the experiences result in reduced social interaction and lost opportunities, both leading to loneliness (see Figure 2). Decreased social interaction was preceded by fear of social situations, social exclusion, stigma, time to be spent with the relative, embarrassment, and losing honor, which in turn could lead to loneliness. Aspects of lost opportunities (inability to get married and loss of employment) were found to contribute to loneliness. Caregiver gains included more personal strength, compassion for others who experienced the same, focusing on life's positives, and reinvention of the self, which all resulted in a sense of meaning.

## Discussion

This study adds to the existing empirical work on caregivers’ experiences by considering the context of South India and how family structures and stigma influence caregivers’ life experiences and decisions. In particular, we described the changing experiences of caregivers over time in a phase-based model.

Embarrassment and losing honor have been described in the context of mental illness as a factor of burden among caregivers. The relative's embarrassing behavior has commonly been identified as a source of losing honor (Awad & Voruganti, 2008; Chang & Horrocks, 2006; Thara et al., 2003). Caregivers in this study uniquely identified wandering, which caregivers perceived as causing gossip amongst neighbors about lack of care, as a source of embarrassment and losing honor, as well as the perception that the relative does not comply with social expectations of education, employment, and marriage. A study by Thara and Srinivasan (1997) showed a marriage rate of 70% among persons with schizophrenia in TN over a 10-year period, which is high compared to studies conducted in western settings. Pinto (2011) described the fraught relationships between families and women with mental illness, and in particular scrutiny of families related to love and marriage. Both studies indicate the importance of marriage within families, as was conveyed by caregivers in this study; related to both attributing mental illness to marriage troubles, as well as considering the unmarried status of their relative a failure. The high importance given to fulfilling societal expectations in some cases trumps the needs of the individual (Pinto, 2011; Thara & Srinivasan, 1997).

Courtesy stigma was observed to affect family members across the world. Studies in China (Hsaio et al., 2017) and Sweden (Östman & Kjellin, 2002) and a review of studies among caregivers of persons with bipolar disorder (van der Voort et al., 2007) revealed the presence of courtesy stigma. Public stigma (Corrigan & Miller, 2004) was experienced by caregivers in the form of difficulties in finding a spouse, branding the entire family as sinners, stigma in interpersonal interactions, and disintegration of social connections (Angermeyer et al., 2003). Studies on stigma conducted in India mostly focus on the effects on people with mental illness, instead of the stigma experienced by families (Koschorke et al., 2014; Loganathan & Murthy, 2008; Loganathan & Murthy, 2011). We found that courtesy stigma affected families in various ways: stigma related to cultural beliefs about causes of mental illness caused loss of relationships, the inability to participate in social events, and cessation of visits by family members and friends. Some family members lost their employment, which created great financial hardship.

Another aspect of stigma was a sense of loss of honor by families, since the relative with mental illness did not comply with societal expectations, in particular with regards to the inability of the person with mental illness and certain caregivers to get married. Since marriage is an important aspect of the expected life path of both men and women in India, the inability to marry caused great distress to caregivers, which has not been prominently described in studies in high-income countries.

Loneliness as a result of reduced social interactions was an important theme shared by the participants, which is consistent with other literature (Chan, 2011). Reduced social support networks have been shown to increase the burden on caregivers (Brijnath, 2014; Chan, 2011; Chen & Greenberg, 2004; Chien & Norman 2009; Grandón et al., 2008). Unlike findings from studies in South Africa and Botswana (den Hertog & Gilmoor, 2016; Seloilwe, 2006), which showed that family and community members often share care for a person with mental illness, caregivers in this study mostly lacked such support. This could be related to an increase in nuclear families and a decline of extended family ties in India (Chadda & Deb, 2013).

We did not find that stigma directly led to lost opportunities (except when related to marriage); instead, these were a result of the demands of caregiving and the time and resources required to provide treatment and care for the relative with mental illness, which is similar to findings in North India among caregivers of people with dementia (Brijnath, 2014). The acceptance of the caregiver role by husbands in this study is not the norm. Abandonment of wives upon the onset of mental illness is common across India, as is remarriage by men after women become homeless (Gopikumar, Easwaran, et al., 2015).

Caregivers identified aspects of posttraumatic growth, which, as expected, focused more on family relationships and needs of others than on the development of the individual. Accordingly, “compassion,” entailing empathy for others in the same situation and the willingness to help, was most prominent in the caregivers’ stories. In addition, earlier untapped “personal strength” was developed as a result of the caregiver experience. Together with love for their relatives, personal strength provided an important resource for perseverance and to carry out familial duties. Personal strength mostly did not lead to a process of “reinventing the self,” unlike findings in western settings. Lack of focus on “effortful reinvention of self” can also be explained by the collectivist nature of society in Tamil Nadu, in which the larger focus on the needs of family members is deemed more important than individual development (Bauer et al., 2012; Chen & Greenberg, 2004; Shakespeare-Finch & Copping, 2006).

## Conclusion

The findings in this study of caregiver experience in India suggest new avenues for further longitudinal research, in order to develop and test support structures that address the main issues (fear, stigma, and loneliness) and to prevent lost opportunities as a result of caregiving. The extent to which new social relationships (e.g., in the context of helping others, or peer support structures) can contribute to a greater sense of meaning and can counterbalance loneliness needs to be further investigated. While this study was conducted with caregivers who have continued to provide care, understanding the lived experiences of caregivers who have stopped providing care would be informative, in order to devise strategies to prevent the collapse of care by family members. Also, while lack of access to mental health care is a major concern for many families of persons with mental illness, particularly in rural areas of India (Gupta & Sagar, 2018; Jacob, 2017), the sample we studied had accessed the services of an NGO. Further work is needed to understand caregiver experience in situations where services cannot be accessed.

Providing care for a person with mental illness has the potential to alter the course of the caregiver's life drastically, including lost opportunities and reduced social interaction, resulting in loneliness. The consequences of lost opportunities can be particularly profound for families living in poverty, including loss of employment, housing, and inability to marry. Nevertheless, the opportunity to help others, love, and strong family ties have the potential to create positive experiences for caregivers and support structures can therefore be designed to strengthen these areas.

## Notes

1. NALAM community workers are laypersons from the community who have received six months’ training in mental health care and social entitlement facilitation and are employed by The Banyan to address mental health in assigned villages around their residence.

2. Dedoose qualitative analysis software. www.dedoose.com

3. Below Poverty Line certification is provided to households with incomes of INR 27,000 (INR 2,250 per month) or less per annum (Source: Government of India – Ministry of Electronics and Information Technology. https://digitalindia.gov.in/content/below-poverty-line-certificate).
